# Neuroprotective Effects of Moderate Hypoxia: A Systematic Review

**DOI:** 10.3390/brainsci13121648

**Published:** 2023-11-27

**Authors:** Viktoria Damgaard, Johanna Mariegaard, Julie Marie Lindhardsen, Hannelore Ehrenreich, Kamilla Woznica Miskowiak

**Affiliations:** 1 Neurocognition and Emotion in Affective Disorders (NEAD) Centre, Copenhagen Affective Disorder Research Centre, Psychiatric Centre Copenhagen, Frederiksberg Hospital, Hovedvejen 17, DK-2000 Frederiksberg, Denmark; viktoria.damgaard.01@regionh.dk (V.D.); johanna.mariegaard@regionh.dk (J.M.);; 2 Department of Psychology, University of Copenhagen, Øster Farimagsgade 2A, DK-1353 Copenhagen, Denmark; 3 University of Göttingen, 37075 Göttingen, Germany; hannelore.ehrenreich@web.de; 4 Clinical Neuroscience, Max Planck Institute for Multidisciplinary Sciences, City Campus, 37075 Göttingen, Germany

**Keywords:** neuroplasticity, cognition, neurological functions, hypoxia

## Abstract

Emerging evidence highlights moderate hypoxia as a candidate treatment for brain disorders. This systematic review examines findings and the methodological quality of studies investigating hypoxia (10–16% O_2_) for ≥14 days in humans, as well as the neurobiological mechanisms triggered by hypoxia in animals, and suggests optimal treatment protocols to guide future studies. We followed the preferred reporting items for systematic reviews and meta-analysis (PRISMA) 2020. Searches were performed on PubMed/MEDLINE, PsycInfo, EMBASE, and the Cochrane Library, in May–September 2023. Two authors independently reviewed the human studies with the following tools: (1) revised Cochrane collaboration’s risk of bias for randomized trials 2.0; (2) the risk of bias in nonrandomized studies of interventions. We identified 58 eligible studies (k = 8 human studies with N = 274 individuals; k = 48 animal studies) reporting the effects of hypoxia on cognition, motor function, neuroimaging, neuronal/synaptic morphology, inflammation, oxidative stress, erythropoietin, neurotrophins, and Alzheimer’s disease markers. A total of 75% of human studies indicated cognitive and/or neurological benefits, although all studies were evaluated ashigh risk of bias due to a lack of randomization and assessor blinding. Low-dose intermittent or continuous hypoxia repeated for 30–240 min sessions, preferably in combination with motor-cognitive training, produced beneficial effects, and high-dose hypoxia with longer (≥6 h) durations and chronic exposure produced more adverse effects. Larger and methodologically stronger translational studies are warranted.

## 1. Introduction

Cognitive impairment is a key phenotype in a range of disorders of the central nervous system (CNS). Currently, treatment strategies with robust efficacy and long-lasting effects on cognition are essentially lacking [1]. This lack of treatments is partially due to limited insights into the neurobiological processes involved in brain health and disease. Neuroplasticity refers to the capacity of the brain to generate new synapses, dendritic spines and, in general, to upgrade and change brain connectivity in response to learning [2]. These processes play a key role in adaptive CNS functioning and their disruption is associated with impaired cognitive and neurological functioning [3]. Uncovering the mechanisms involved in neuroplasticity is therefore crucial to improving CNS disorders.

The body’s ability to adapt to changes in oxygen levels is an evolutionary trait, but we are only beginning to understand the response of the CNS to lowered levels of ambient oxygen, also referred to as inspiratory hypoxia [4]. Hypoxia can consist of continuous (constant) or intermittent (cyclical, intervaled by brief periods of normoxia) stimuli with repeated (i.e., daily) or chronic exposure [5]. A common layperson’s perception has been that hypoxia is exclusively associated with negative effects on the CNS. In contrast, recent emerging evidence indicates that *moderate* doses of hypoxia (typically shorter intermittent or continuous repeated sessions of 10–16% O_2_ exposure over longer time periods (typically several weeks)) has possible therapeutic effects [6]. Specifically, a small pilot study exploiting intermittent hypoxia (eight cycles of five minutes of 10% O_2_ exposure with normoxia in between, repeated three times weekly for eight weeks) reported improved cognitive performance in seven elderly patients with amnestic mild cognitive impairment [7]. In line with this, another small study in 34 geriatric participants demonstrated improvements in cognitive and motor performance following 35–45 min of intermittent hypoxic (12% O_2_) and hyperoxic (30% O_2_) breathing repeated 2–3 times weekly for 5–6 weeks [8,9]. Remarkably, continuous hypoxia (11% O_2_) has also been associated with extending lifespan by 50% and delayed onset of neurological dysfunction in a mouse model of aging [10].

In contrast, studies investigating the effects of *acute* and *severe* doses of hypoxia (typically intensities of ≤9% O_2_) exposure consistently showed impaired cognitive performance in humans [11,12,13] and inhibited neuroplasticity, including neuronal apoptosis and increased oxidative stress, in animal models [14,15]. This apparent distinctive effect of hypoxia was also noted in a recent narrative review by Navarette-Opazo and Mitchell, where moderate intermittent hypoxia (9–16% O_2_) exposure with low cycle numbers produced mainly beneficial effects as opposed to severe intermittent hypoxia (3–8% O_2_ with more cycles), which produced mostly adverse effects across human and animal studies [6]. Taken together, this suggests that the balance between potential therapeutic vs. pathogenic effects of hypoxia depends on dose (i.e., frequency, duration, and intensity (level of hypoxia) per session as well as the length of the intervention) and exposure type (hypobaric vs. normobaric or intermittent vs. continuous). Nevertheless, there is a general lack of consensus regarding experimental procedures across hypoxia studies, resulting in many inconsistent findings due to variations in oxygen levels, intensity of exposure, and duration per session [6,16] as well as an inconsistent terminology, further complicating the comparability of efficacy across studies [5]. Given this, there is a need for systematic investigations of these moderate hypoxia interventions to determine the optimal intervention schedule.

The objective of this systematic review was to assess the effects of moderate intensities of hypoxia (i.e., 10–16% O_2_) exposure for longer periods (≥14 days) (i) on cognitive or neurological outcomes in humans and the methodological quality of these trials, and (ii) on measures of neuroplasticity and cognition in animal studies, to determine optimal treatment protocols as a guideline for future interventional studies in the field.

## 2. Methods

### 2.1. Data Source

This systematic review followed the procedures of the preferred reporting items for systematic reviews and meta-analysis (PRISMA) 2020 statement [17]. A comprehensive systematic computerized search was conducted on the PubMED/MEDLINE, PsycInfo, and EMBASE and Cochrane Library databases from May to September 2023. The search profile involved the four elements, “Hypoxia”, “Cognitive, neurological, and neuroplasticity-related outcome”, “Intervention”, and “Exclusion criteria”, with each of their combinations and alternative key words in the respective databases (see Supplementary Material for details on the search profile). A protocol of the review was registered on the online database PROSPERO (registration number: CRD42023469082).

### 2.2. Selection Criteria

The search criteria were defined in line with the population, intervention, comparison, and outcome (PICO) framework. The research question was: In both humans and animals (population), can moderate hypoxia exposure interventions (intervention), when compared to a normoxia control condition (comparison), affect cognitive and neurological function and markers of neuroplasticity (outcome)?

We included only original peer-reviewed empirical reports involving in vivo investigations of moderate hypoxia interventions on the brain. Eligible reports involved (a) both healthy individuals and individuals with clinical conditions, and animal subjects; (b) a longer intervention (≥14 days) of continuous or intermittent hypoxia of moderate intensity (as defined by O_2_ levels in the range of 10–16 ± 0.5% [6]), which could be normobaric (simulated altitude) or hypobaric (real altitude), which could also include combinations with other changes in O_2_ levels (i.e., hypoxia-hyperoxia treatment) as well as pharmacological or behavioral combined interventions to investigate potential synergistic effects; (c) parallel group studies or cross-over studies involving a normoxia control condition; (d) objective measure of cognitive and neurological function, including motor function, neuropsychiatric behavior, structural and functional neuroimaging, and objective neurobiological markers of brain plasticity, including neuronal morphology and myelination, oxidative stress, inflammation, erythropoietin, neurotrophins, and markers of Alzheimer’s pathology; and (e) articles published in English only. We excluded articles that investigated the effects of (I) acute hypoxia (less than 14 days); (II) severe or mild intensities of hypoxia interventions (≤9% or ≥17% O_2_); (III) had no normoxia control condition or did not include any hypoxic intervention (i.e., cross-sectional comparisons between high altitude vs. sea level populations); (IV) were written in languages other than English; and (V) were reviews, meeting abstracts, dissertations, and case reports.

### 2.3. Study Selection

Two authors (V.D. and J.M.L.) independently conducted a primary title/abstract screening for possible eligible studies and, following this, a secondary full-text screening using the Covidence systematic review software 2023, Veritas Health Innovation, Melbourne, Australia (www.covidence.org). Additional hand searches were further performed by tracking and screening citations in the included articles to ensure inclusion of all relevant reports. In all phases, articles were considered in accordance with the inclusion and exclusion criteria. Interrater reliability was measured as percentage agreement and was calculated as the number of agreements divided by the total number of screened references. Agreement between the two authors was high (primary screening: 97%; secondary screening: 96%). Disagreements were discussed and consensus was reached in all cases through discussions with a third author (KWM). Two authors (V.D. and J.M.) extracted the measures of interest and presented results from the eligible reports in Table 1 and Table 2. The measures of interest were predefined in accordance with the objective of the review and included the following: author and year of publication, study design, population and sample characteristics, comparison groups, duration, and type of hypoxia intervention, O_2_ level, outcome measures, and main findings. If data were missing, they were defined as ‘no information’. The syntheses of the included reports were predefined according to human studies (Table 1) and animal studies (Table 2), respectively.

### 2.4. Risk of Bias Assessment

The risk of bias within and across the included human reports was assessed by two authors (V.D. and J.M.). For interventional studies, risk of bias was evaluated in accordance with the revised Cochrane collaboration’s risk of bias 2 (RoB 2, 2021 version) tool (https://www.riskofbias.info/welcome/rob-2-0-tool/rob-2-for-crossover-trials, accessed on 3 October 2023). For case–control studies, risk of bias was assessed with the risk of bias in non-randomized studies—of interventions (ROBINS-I) tool [74]. Risk of bias evaluations were independently conducted by the two authors, and disagreements were discussed with a third author (KWM). If any information was missing in the included articles, additional searches for the registered studies on clinicaltrials.gov were performed, and a search for published study protocols were conducted on relevant search engines. Table 3 and Table 4 display the risk of bias evaluations for the included human studies. The PRISMA 2020 checklist was completed (see Supplementary Material).

## 3. Results

**Figure 1:**
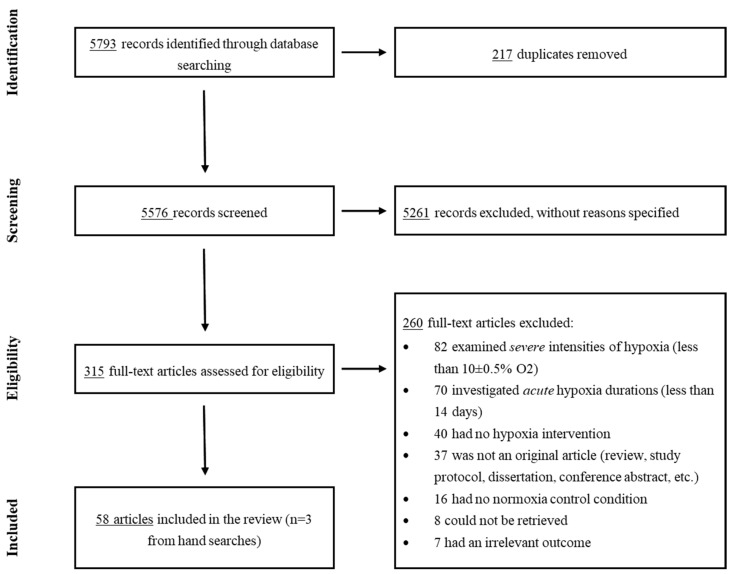
PRISMA 2020 Flowchart.

The systematic search, together with additional hand searches, identified 5582 articles (after removal of duplicates), and all these were included for the title/abstract screening (primary screening). Out of these, 315 articles were further evaluated for eligibility through a full-text reading (secondary screening). This resulted in 58 articles that met the inclusion criteria and were thus included in this review (Figure 1 depicts the PRISMA 2020 flowchart). Of these, ten reports were on humans (N = 274 individuals), and the remaining 48 studies were on animal models. Table 1 and Table 2 display the characteristics of the included studies investigating the effects of moderate hypoxia in human and animal subjects, respectively.

## 4. Effects of Moderate Hypoxia in Human Studies

Of the eight human studies, six (75%) observed largely beneficial effects of intermittent moderate hypoxia (i.e., 10–14% O_2_ or 80% SpO_2_) or hypoxia-hyperoxia repeated for 30–90 min sessions for 3–7 weeks on all or most cognitive and/or neurological outcomes in healthy participants, geriatric participants, and patients with mild cognitive impairment [8,9,19,22,23,24,25]. One study (12.5%) in patients with traumatic brain injury reported no changes in any outcome after continuous 10–14% O_2_ for 30 min to two hours repeated over 5–12 weeks [21]. Finally, one study (12.5%) in college students found negative effects after two years of chronic high-altitude living (3658 m corresponding to approx. 13% O_2_) [20].

### 4.1. Cognition

Eight studies investigated the effects of hypoxia exposure on cognition (three reports had 100% overlapping samples and are therefore counted as one study here) [8,9,18]. Of these, six studies examined global cognitive functioning assessed by either a comprehensive test battery [21] or a screening tool [9,19,22,24,25]. Two studies with N = 25–34 geriatric patients reported improved global cognition after relatively similar treatment protocols, involving normobaric, intermittent hypoxia-hyperoxia breathing (3–30 cycles of 10–14% and 30–40% O_2_ for 30–45 min repeated 2–3 times weekly for 5–6 weeks) combined with physical training compared to normoxic breathing and physical training [18,19]. Two studies of N = 28 mild cognitive impairment patients observed global cognitive improvement following normobaric intermittent hypoxia-hyperoxia (12% and 33% O_2_ for 15 min repeated five times per week for three weeks) compared to normoxia controls [24,25]. Two studies observed no effect of hypoxia on global cognition [21,24]. One of these was of N = 9 patients with traumatic brain injury, who underwent two hours of continuous approx. 12% O_2_ repeated for 12 weeks in addition to muscle electrostimulation [21]. The other study was of N = 34 older adults after normobaric intermittent hypoxia (80–90% SpO_2_) breathing for 15 min repeated five times per week over four weeks [22].

Three studies specifically investigated the effects of hypoxia on attention and processing speed [20,21,22]. One study of N = 34 older adults reported improved attention, but no group-by-time change in processing speed, after six weeks of combined normobaric, intermittent hypoxia (90–80% SpO_2_ for one hour repeated three times weekly for six weeks) with a full body strength–endurance training program [22]. One study with N = 9 patients with traumatic brain injury detected no difference in processing speed after two hours of hypobaric, continuous approx. 12% O_2_ repeated three times per week over six weeks combined with muscle electrostimulation [21]. Another case–control study comparing N = 49 college students moving to high altitude (hypobaric, chronic exposure to 3658 m corresponding to approx. 13% O_2_) with N = 49 college students living at sea level found no group-by-time difference in processing speed, but a within-group decline in students who lived for two years at high altitude [20]. Nevertheless, students (N = 98) were allocated to high altitude vs. sea level groups based on their a priori acceptance to a college located at either high altitude or sea level, yielding bias in the randomization process in this study (see Table 4 for risk of bias evaluation).

Two studies further examined the effects of hypoxia on verbal memory [20,21]. One study observed no change after hypobaric continuous 12% O_2_ for two hours repeated three times weekly for six weeks in addition to muscle electrostimulation for N = 9 patients with traumatic brain injury [21]. Accordingly, the other study comparing college students living at high altitude (hypobaric approx. 13% O_2_ chronically for two years) vs. sea level showed no difference in verbal memory performance, although there was a within-group effect showing verbal memory decline in the chronic high-altitude group [20].

Finally, two studies investigated the effects of hypoxia interventions on executive functioning [21,23]. One study in older adults comparing hypoxia and aerobic training (N = 17) with normoxia and aerobic training (N = 16) found no significant difference in executive functioning, but a within-group improvement in the hypoxia and aerobics group (normobaric hypoxia consisted of four weeks of intermittent 90–80% SpO_2_ for 90 min repeated three times per week) [23]. The other study with N = 9 patients with traumatic brain injury found no change in executive functioning after two hours of continuous hypobaric 12% O_2_ for six weeks in addition to muscle electrostimulation compared to a normoxia control group [21].

Taken together, these findings suggest that smaller doses of normobaric intermittent hypoxia (with moderate intensities between 10 and 14% O_2_) applied for 30 to 90 min sessions enhance cognitive performance [18,19,22,23,24], whereas higher doses of hypoxia (e.g., 12% O_2_ continuously applied for two hours over 12 weeks or continuous high-altitude exposure chronically for two years) may not change cognition [21] or negatively impact cognition [20].

### 4.2. Motor Function

One study of N = 34 geriatric patients investigated the effect of normobaric intermittent hypoxic-hyperoxic (10–14%–30–40% O_2_) breathing for 3–9 cycles over 30–45 min combined with multimodal training repeated 2–3 times per week over 5–6 weeks on motor function in three reports (100% overlapping sample) [8,9,18]. While patients in the hypoxia-hyperoxia group exhibited improved performance on a walking test compared to normoxia, no change was detected in the remaining three tests of motor function and mobility [8,9,18]. The study thus suggests a positive effect of intermittent hypoxia exposure on some aspects of motor function.

### 4.3. Neuroimaging

Three studies investigated the effects of hypoxia on the brain with neuroimaging methods [20,24,25]. Two similar studies with N = 28 patients with mild cognitive impairment and N = 13 healthy participants investigated the effect of normobaric, intermittent hypoxia-hyperoxia (15 min of cycles of 12% and 33% O_2_ five times per week repeated for three weeks) with electroencephalography (EEG) and found decreased P300 and N200 latencies, indicative of improved attention, working memory, and sensory processing, along with improved global cognition in the hypoxia group compared to normoxia controls [24,25]. One case–control study examined the effects of N = 49 students moving to study at a college at high altitude (3658 m corresponding to hypobaric continuous 13% O_2_ chronically for two years) vs. N = 49 students living at sea level on brain structure using structural magnetic resonance imaging (sMRI) [20]. Specifically, the chronic high-altitude group presented with decreased caudate grey matter volume compared to the control group, which correlated with poorer verbal memory performance [20]. Further, there was a within-group change in fractional anisotropy values in multiple white matter tracts in the high-altitude group, with both increases in thalamic and fronto-occipital regions and decreases in temporal regions [20]. While these neuroimaging findings are conflicting, it suggests that lower doses of, e.g., intermittent hypoxia interventions can improve brain functioning and associated cognitive performance, while higher doses, e.g., more chronic exposure at high altitude, may negatively affect structural brain measures.

### 4.4. Inflammation

One study investigated the effects of normobaric intermittent hypoxia-hyperoxia (four cycles of 12% and 33% O_2_ repeated five times weekly for three weeks) on markers of inflammation in N = 27 patients with mild cognitive impairment and healthy participants [25]. The study showed increased levels of inflammation following hypoxia compared to normoxia. This finding was interpreted as potential adaptive reprogramming, generating therapeutic effects against neuropatological changes, given the observed parallel cognitive improvement following hypoxia [25].

## 5. Risk of Bias Evaluations

Table 3 presents the RoB 2 evaluations for the nine reports on controlled human trials included in the review. All studies were evaluated to be of ‘high risk’ in the overall assessment. The primary concerns of the methodology in these studies regarded inadequate information on (or a complete lack of) randomization (k = 5), suboptimal analyses without intention-to-treat statistical protocols (k = 9), and a lack of (information on) blinding in the outcome assessments (k = 6). Table 4 displays the ROBINS-I evaluation for the single included observational study. This study was found to be of ‘serious risk of bias’ due to a lack of adjustment for important confounders, possible selection biases in the included participants, and a lack of information on assessor blinding [20]. Overall, the included human studies suffered from several methodological challenges according to the Cochrane guidelines, yielding a high risk of bias, which indicates that the study of hypoxia effects on cognition is still in its infancy and that the findings must be considered vague and very preliminary.

## 6. Effects of Moderate Hypoxia in Animal Studies

Of the 48 included animal studies, 28 (58.5%) observed adverse effects of hypoxia exposure [27,28,29,31,32,35,36,38,39,40,41,46,47,49,50,57,59,61,62,63,64,66,67,69,70,71,72], while 18 studies (37.5%) reported beneficial effects [26,33,34,37,42,43,44,45,48,51,53,55,56,58,60,65,68,73] and two studies (4%) found either no change in outcomes or mixed findings in both directions after hypoxia treatment [30,52].

### 6.1. Cognition and Neuropsychiatric Behavior

Thirty-eight studies investigated the effects of moderate hypoxia exposure on cognitive functioning and anxiety- and depression-like behavior in rodents (see Table 2). Thirty-three studies examined hypoxia-related changes in hippocampal-dependent learning and memory performance, mostly assessed with the Morris water maze paradigm (27 of 33 studies) [30,31,32,34,35,36,38,39,40,41,42,44,45,50,51,54,55,56,57,59,60,61,62,63,64,65,72]. Of these, 21 studies reported *impaired* learning and memory following various protocols with higher doses of hypoxia, including normobaric intermittent hypoxia (mainly 10% O_2_) chronically administered for +10 h daily over 2–4 weeks, or hypobaric continuous hypoxia of 11–13% O_2_ chronically administered over 4–12 weeks [31,32,35,36,38,39,40,41,50,54,57,59,61,62,63,64,66,67,69,71,72]. In contrast, nine studies, in which study designs predominantly included shorter, continuous hypobaric hypoxia exposures for four to six hours daily, found *improved* learning and memory (intensities between 10.8% and 16% O_2_ repeated for 14–28 days) [34,42,44,45,55,56,60,65,68]. Finally, two studies observed no difference in learning and memory following nomobaric intermittent 10% O_2_ for 21–30 days vs. normoxia controls [30,51].

Seven studies further investigated the effects of hypoxia on working memory, using working-memory versions of maze paradigms and object recognition paradigms [38,49,56,60,61,64,70]. Of these, four detected impaired working memory performance following both hypobaric or normobaric hypoxia, involving continuous exposures of 10.5–13.5% O_2_ chronically for 8–24 h daily over 2–4 weeks *or* intermittent 10% O_2_ exposure chronically for two weeks [38,49,63,70]. In contrast, two studies found improved performance following hypobaric continuous 14% O_2_ for 6 h repeated daily for 28 days [56] or normobaric continuous 11% O_2_ chronically for 28 days [60]. Finally, one study observed no difference in working memory after intermittent 10% O_2_, repeated for eight hours daily for 14 days [61].

Finally, 11 reports studied hypoxia-related effects on anxiety- and depression-like behavior, and locomotor functioning, which was mostly assessed with the open field test paradigm [27,39,44,45,50,57,61,67,68,71,73]. Of these, four studies found reduced anxious and depressive behavior (e.g., more time spent in open arms and physical state of fur) after 4 h daily continuous hypobaric 10.8–16% O_2_ repeated for 2–4 weeks [44,45,68,73]. However, four studies showed no difference in anxiety-related behavior following 2–4 weeks of 10%-16% O_2_, administered both repeated or chronically daily for 4–12 h [27,39,61,67]. Finally, three studies found increased anxiety-like behavior (higher locomotor activity and less time spent in open arms), mainly following intermittent hypoxia chronically for more than 8 h daily with intensities between 10% and 12.3% O_2_ over 14 days to 8 months [50,57,71].

Taken together, likely due to the very heterogeneous study designs and the sometimes questionable quality of the studies, the findings are highly inconsistent. Nevertheless, most studies report impaired cognitive functioning following larger doses of both normobaric and hypobaric hypoxia exposure, e.g., intermittent hypoxia with 10% O_2_ that was chronically administered over several weeks. However, studies exploiting lower doses of continuous and intermittent hypoxia (i.e., shorter durations of repeated frequency and higher O_2_ levels of 14–16%) find beneficiary effects on both learning and memory, working memory, anxiety- and depressive-like behavior.

### 6.2. Motor Function

Seven studies investigated the hypoxia-related effects on motor functioning in rodents [26,37,46,47,48,51,58]. Motor function was evaluated across many paradigms, but the most common was the Rotarod test assessing grip strength and motor coordination [75]. Four studies reported improved motor function following hypoxia exposure compared to normoxia [26,37,48,58]. Specifically, one study found enhanced motor–cognitive learning and enduring performance following three weeks of continuous 12% O_2_ exposure [58]. Two studies demonstrated preserved motor function after hypoxia, one involving intermittent 9.5–10% O_2_ with 5–8 daily cycles repeated for 20 days in models of ethanol withdrawal [37], and the other employing normobaric continuous 11% O_2_ eight hours daily for two weeks in a stroke model [48], when compared to normoxia-treated rodents. One study involving a spinal cord injury model found motor function improvement in one out of three tasks following 12 weeks of intermittent 11% O_2_, administered for 50 min sessions and repeated four days per week, with concurrent task-specific training [26]. In contrast, two studies observed impaired motor performance following 25–28 days of normobaric continuous 10–11% O_2_ exposure, either administered chronically for 12 [27] or 24 [46] hours daily, although this negative effect was restricted to one out of two motor tasks in one study [28]. The remaining study reported no change in motor function after six hours daily continuous 10% O_2_ repeated over 21 days [47]. Taken together, these findings suggest the possible beneficial effects of intermittent or continuous hypoxia with intensities of 11–12% O_2_ with repeated exposures over 14–21 days on motor performance and recovery in animal models.

### 6.3. In Vivo Neuroimaging

Four studies studied the effects of hypoxia on the brain using in vivo neuroimaging approaches in animals [29,64,70,71]. One EEG study in rats found reduced theta and delta activity following hypobaric, continuous 14% O_2_ chronically for 28 days [29]. This was hypothesized to be related to poorer cognitive performance, although this study did not include any direct measures of cognition. Another EEG study in mice found decreased P300 amplitude following hypobaric, continuous 12.5–13.5% O_2_ administered chronically for 14 days in addition to a reduction in working memory performance [70]. An fMRI study in rats found decreased resting-state activity after hypobaric continuous hypoxia administered chronically for 4 weeks (10% O_2_) alongside impaired memory [64]. Finally, a structural MRI study showed reduced hippocampal volume in rats subjected to continuous high altitude living (4250 m corresponding to approx. 12% O_2_) chronically for 8 months compared to rats living at sea level, which correlated with impaired memory performance [71]. These studies thus indicate the potentially negative effects of higher doses of hypobaric hypoxia or high-altitude exposure on cognition-related brain imaging measures in rodents.

### 6.4. Neuronal Morphological Changes

Twenty-three studies examined hypoxia-related changes in neuronal morphology in rodents (see Table 2). Out of these, 13 studies investigated neurodegeneration, which involved apoptosis in the hippocampus [30,35,36,40,61,62,69,71,73], frontal and temporal cortex [43,70], and the basal forebrain [57]. Eight studies reported increased neuroapoptosis following higher hypoxic doses, involving 8+ hours of daily normobaric or hypobaric hypoxia (intensities between 10 and 13% O_2_) chronically for 2–8 weeks when compared to normoxia controls [35,36,40,57,62,69,71,72]. Two studies found no apoptosis, one after normobaric intermittent 10% O_2_ exposure chronically for one month [30], and one after hypobaric continuous 11–14% O_2_ repeated for four hours daily for two weeks [73]. One study found prevented neuroapoptosis in Alzheimer’s disease rats treated with hypobaric continuous hypoxia for 4 h daily (12.5% O_2_) repeated for two weeks compared to normoxia treatment [43].

Eight studies investigated neuroregenerative processes following hypoxia exposure in rodents, including hippocampal neurogenesis [28,33,45,55,56,73] and long-term potentiation [42,68]. Of these, seven found *enhanced* neurogenesis or long-term potentiation exploiting different protocols of low-dose hypoxia, primarily hypobaric continuous hypoxia of 11–16% O_2_ repeated for 4–6 h daily for 2–4 weeks [33,42,45,55,56,68,73]. The remaining study found *decreased* neurogenesis after normobaric, continuous 10% O_2_ chronically for two weeks [28].

Furthermore, five studies investigated hippocampal synaptic morphology [41,54,56,61,68]. Two studies found increased synaptic plasticity following 4–6 h repeated hypobaric continuous hypoxia (10.8–14.2% O_2_) for four weeks [56,68]. In contrast, two studies reported synaptic loss after intermittent or continuous hypoxia for 6–8 h repeated daily with intensities between 10 and 11.1% O_2_ [41,61]. The final study observed no change in synaptic morphology after 12 weeks of hypobaric, continuous 11% O_2_ chronic exposure [54].

Finally, two studies investigated changes in dendritic morphology [38,56]. One study found increased dendritic plasticity after continuous hypobaric hypoxia (6 h daily at 14.2% O_2_) repeated for one month [56], whereas one study reported decreased dendritic branching following intermittent 10% O_2_ exposure with 240 cycles for 12 h per day for 20 days, although this effect was only observed in male, and not female, rats [38].

Taken together, the findings from these animal models suggest that hypoxia exposure mostly has a positive impact on neuronal morphological changes in the brain, although findings vary with the *direction* of these changes seemingly being dependent on the dose and intensity of exposure, with lower doses producing most consistent beneficial effects.

### 6.5. Myelination

Four reports studied hypoxia-related changes in myelination, including myelinogenesis and degeneration and levels of mature myelin [27,28,46,54]. Three found reduced myelination after chronic exposure to normobaric continuous hypoxia (10–11% O_2_) for 2–4 weeks [27,28,46]. One study did not observe any change in myelination after hypobaric continuous hypoxia (chronic 11% O_2_) compared to normoxia [54]. Based on this, higher hypoxic doses involving lower levels of oxygen (<11%) and longer-duration chronic exposure might negatively affect myelination processes.

### 6.6. Neurotrophins

Six studies investigated if moderate hypoxia affects neurotrophin levels, which involve brain-derived neurotrophic factor (BDNF), vascular endothelial growth factor (VEGF), and neurotrophin-3 (NT-3) levels [45,48,51,52,53,73]. All studies reported increased neutrophin levels after varied types of moderate hypoxia protocols, mainly involving either intermittent hypoxia with 10 cycles per day (approx. one hour) repeated for 3–10 weeks, or continuous hypoxia with repeated exposure chronically up to eight weeks. Taken together, these results indicate that both intermittent and continuous moderate hypoxia increases neurotrophin levels.

### 6.7. Erythropoietin

Two studies examined the effects of hypoxia on erythropoietin (EPO) levels, a multifunctional growth factor known for its neuroplastic properties [51,73]. One study in rats reported increased EPO levels in the hippocampus after four hours daily repeated 11% O_2_ exposure (hypobaric continuous hypoxia) over two weeks compared to normoxia controls [73]. In line with this, a study in a mouse model of Alzheimer’s disease found increased cerebrocortical EPO following normobaric intermittent hypoxia with 10 cycles of 10% O_2_ and normoxia, administered for one hour daily repeated over 2–3 weeks, compared to normoxia-treated mice [51]. This suggests that moderate hypoxia exposure can heighten EPO expression.

### 6.8. Neuroinflammation

Five studies in rodents investigated the hypoxia-related changes in neuroinflammation markers, i.e., pro-inflammatory cytokines like interleukin-6 and tumor necrosis factor alpha [46,57,59,65,71]. Of these, four studies found increased levels of neuroinflammation following higher doses of both intermittent and continuous hypoxia (chronic exposure of 10–12% O_2_ for 25 days to eight months) compared to normoxia [46,57,59,65,71]. However, one study demonstrated reduced neuroinflammation following hypobaric continuous hypoxia (14% O_2_) repeated for 4 h daily for 14 days in a mouse model of Alzheimer’s disease [65]. These findings indicate that hypoxia can alter neuroinflammatory markers, with some evidence that higher doses (e.g., with lower intensity levels (10–12%) of O_2_ for longer durations) may increase inflammation, while more moderate doses (e.g., repeated 14% O_2_ for shorter durations) may alleviate inflammation.

### 6.9. Oxidative Stress

Eight studies investigated the effect of hypoxia on levels of oxidative stress in rodents, involving increased lipid peroxidation and decreased antioxidant levels in the brain [31,35,44,52,57,66,71,72]. Seven of these found increased levels of oxidative stress following various protocols, including intermittent (320–480 cycles of 10% O_2_) with chronic exposures for 14 days to 8 months [31,35,52,57,66,71,72]. One study found no significant effect on oxidative stress after hypobaric continuous hypoxia (12.5% O_2_) administered for 4 h daily, repeated for 14 days [44]. These findings indicate that longer-duration and chronic exposures of hypoxia can heighten levels of oxidative stress in the brain.

### 6.10. Markers of Alzheimer’s Disease

Six studies investigated how hypoxia affects Alzheimer’s disease markers, including tau phosphorylation [39,63,66], β-amyloid [41], amyloid plaque [65], and hippocampal vascular density [60]. Four studies reported increased levels of Alzheimer’s disease markers, indicating worsened pathology, following continuous hypoxia (6–8 h daily exposures of 10–11% O_2_) repeated for 2–8 weeks [39,41,63,66]. However, one study of hypobaric continuous hypoxia (14% O_2_ given in four hour sessions repeated for two weeks) demonstrated decreased levels of amyloid plaque in the hippocampus (but not in the cortex) in an Alzheimer’s disease model in mice [65]. Another study found *increased* hippocampal vascular density following normobaric continuous hypoxia with 11% O_2_ chronically for 28 days, which lasted for at least two months after hypoxia treatment [60]. This effect contrasts with decreased vascular density in neurodegenerative conditions such as Alzheimer’s disease. Overall, the conflicting findings indicate that hypoxia can affect Alzheimer’s disease markers, although the *direction* is controversial and possibly dose-dependent.

## 7. Discussion

This systematic review identified 58 articles investigating the effects of moderate hypoxia exposure on cognitive and neurological functions and markers of neuroplasticity across humans and animal subjects. Of these, eight studies were conducted in humans (three articles overlapping in sample) with various CNS conditions like traumatic brain injury and mild cognitive impairment or healthy individuals, whereas 48 studies were conducted in rodents, including models of spinal cord injury and Alzheimer’s disease. In the human reports, six studies (75%) found beneficial effects for hypoxia exposure, while the remaining studies observed either no efficacy (k = 1; 12.5%) or impairment (k = 1; 12.5%). The findings were more variable in the animal studies with around 28 studies (58.5%) demonstrating the negative effects of hypoxia exposure on measures of learning and memory, apoptosis, inflammation, and oxidative stress, while 18 studies (37.5%) reported positive effects, and two studies (4%) showed either no or mixed findings. In general, studies exploiting lower doses of normobaric intermittent or continuous hypoxia exposure with sessions ranging from 30 min to 4 h repeated over 2–12 weeks showed the most consistent beneficial effects, while studies applying higher doses of both normobaric or hypobaric, intermittent or continuous hypoxia with +6 h sessions, chronically administered over 2 weeks to 2 years, showed more consistent negative effects. Indeed, 84% of the ‘low dose’ hypoxia studies (k = 9 in humans; k = 11 in animals) observed beneficial effects on measures of global cognition, memory, attention, motor function, and neuroplasticity markers (i.e., increased levels of neurotrophins, EPO, neurogenesis, etc.) [9,18,19,22,23,24,26,42,43,44,45,53,65,69,73]. In contrast, only 18% of the studies with higher doses of hypoxia exposure (k = 1 in humans; k = 37 in animals) showed beneficial effects, while 79% reported negative effects on cognition, brain structure, oxidative stress, inflammation, and Alzheimer’s disease markers [20,27,28,31,32,35,36,38,39,40,41,47,49,50,54,57,59,61,62,63,64,66,69,70,71,72]. Importantly, the risk of bias was high for all human studies, indicating the very preliminary state of the current research on hypoxia and CNS disorders.

The majority of the human studies found beneficial effects of moderate hypoxia exposure on measures of cognitive and neurological functioning. These positive studies were characterized by similar treatment schedules of low-dose normobaric, intermittent hypoxia involving fewer cycles (i.e., 3–30 cycles) and shorter (i.e., 30–90 min) durations with intensities between 10 and 14% O_2_, and most were in combination with concurrent physical training programs. This suggests that low-dose intermittent and repeated hypoxia training could be an efficacious intervention on the functioning of the CNS and may be particularly efficacious when combined with a motor-cognitive intervention. Indeed, the animal studies that showed beneficial effects of hypoxia involved relatively similar treatment procedures with session durations ranging from 50 min to 4 h, mainly continuous with repeated exposures over 2–12 weeks with moderate intensities between 10 and 16% O_2_, although only one of these studies involved concurrent physical training [26]. Accordingly, mechanistic findings from these animal studies suggest that markers of neuroplasticity (i.e., neuronal and synaptic growth, increased neurotrophins and EPO levels in the brain) are targeted by physiological manipulation of oxygen and may underlie the observed effects on cognitive and neurological functioning across animals and humans. Taken together, this indicates that lower doses of both intermittent and continuous hypoxia training with moderate O_2_ levels and repeated short sessions could be an effective intervention targeting CNS disease by stimulating neuroplasticity markers, with potentially synergistic effects arising from concurrent training as it exploits the neuroplastic potential generated by the hypoxia exposure. Nevertheless, these findings are still preliminary due to the high risk of bias and small sample sizes, and future large-scale randomized controlled trials are thus highly warranted to assess the potential neuroprotective effects of moderate hypoxia exposure.

Regardless of the most consistently positive effects of hypoxia interventions in the human studies, it is noted that most of the animal studies (56%) showed adverse effects. Possible mechanisms for these observed negative effects involve increased neuroapoptosis, heightened oxidative stress, increased Alzheimer’s disease markers, and inflammation in the brain as evident from the preclinical studies. There were some commonalities across these studies. Firstly, animal subjects often live in impoverished conditions, and it was not clear if animals from the included studies had access to enriched environments or were deprived of environmental stimulation during the studies [76]. This could have impeded the optimal physiological effects of hypoxia on neuroplasticity. Indeed, studies in which animals were living in environmentally enriched conditions, such as access to running wheels [58] or physical training [26] showed beneficial effects of hypoxia. The seeming lack of environmental enrichment on most animal hypoxia studies contrasts with the human studies in two ways: (i) firstly, human participants are *not* subjected to impoverished conditions but live their normal enriched lives during trial participation, and (ii) the human hypoxia interventions mostly (78% of studies) combined hypoxia exposure with other activities such as physical exercise [8,9,18,19,22,23,24]. Therefore, it is possible that physical activity and enriched environments are crucial for maximizing the neuroplastic potential generated by hypoxia exposure, leading to cognitive improvements [77]. More generally, this discrepancy between animal and human studies illustrates the limitations of using animal models to investigate the functioning of the human CNS [78], which may contribute to the sometimes poor predictive validity of treatment effects in animal models for efficacy on CNS disorders in humans [79].

Further, most studies observing negative effects, including a majority of the animal studies, employed higher doses of hypoxia (i.e., *longer* sessions of continuous or intermittent hypoxia repeated from +6 h daily to also chronic exposure, e.g., moving to high altitude or 12 h daily). This provides insights into the optimal dosing and timing of hypoxia interventions, pointing to more positive effects from normobaric hypoxia and lower doses (i.e., shorter, continuous or intermittent hypoxic sessions for less than 4 h, repeated 3–5 times weekly or daily) and multimodal interventions. This is in line with the concept of *hormesis* [80], which stipulates that the body should be exposed to small doses of stress (e.g., hypoxia) in order to compensate in a beneficial way, i.e., by triggering markers of neuroplasticity. In contrast, higher hypoxic doses (i.e., too severe O_2_ intensities, and too long or chronic exposure) may result in excessive stress for the system, potentially explaining the observed negative findings in studies employing hypoxia for longer durations. Indeed, studies have generally shown that severe intensities (less than 10% O_2_) of hypoxia have a negative impact on the brain [81,82,83]. Further, commonly employed ‘chronic intermittent hypoxia’ models of sleep apnea with intermittent 10% O_2_ exposure administered chronically over several weeks have also produced consistent neuropathogenic effects [84]. On the contrary, it is also relevant to speculate whether even milder forms of hypoxia may be sufficient, although underdosing at, e.g., 17% O_2_ is also unlikely to generate the desired effects [85]. Nonetheless, some studies exploiting even shorter periods of moderate hypoxia interventions with 10–16% O_2_ but for less than 14 days found beneficiary effects on executive functioning in healthy individuals [86], walking abilities in patients after spinal cord injury [87], and alleviated memory impairment in a rat model of stroke [88], although these were not included in the present review. Taken together, this suggests that the optimal intensity and dosing of hypoxia for exploiting neuroplastic benefits are likely to be moderate around 10–16% O_2_ with shorter intermittent or continuous types of exposures involving 30 min to 4 h sessions, with repeated frequency +3 days weekly over 2–6 weeks.

A limitation of this systematic review is that it did not include a quantification of the effects of the hypoxia interventions through meta-analysis. However, there were several issues with conducting a meta-analysis on the studies included in this review. Firstly, the research field is at a very early stage as reflected by the high risk of bias across the human studies. Secondly, there was high heterogeneity in the treatment schedules and outcome measures, which would complicate the comparison of the effects across studies. Another limitation was the predefined inclusion criteria of solely moderate intensities of hypoxia (oxygen levels in the range of 10–16%) and minimum duration of 14 days with no upper cut-off for duration. These criteria are somewhat broad and yield heterogeneous treatment protocols. Nevertheless, the aim of this review was to provide an umbrella perspective on this emerging field and provide insights into the potential optimal treatment schedules of hypoxia training that can guide future interventional studies. Finally, the translational approach comparing clinical and preclinical studies further allows for deeper mechanistic insights of moderate hypoxia interventions.

In conclusion, emerging translational evidence suggest that lower doses of moderate hypoxia exposure can improve aspects of cognitive and neurological functioning and markers of neuroplasticity, including learning and memory, motor abilities, neuronal and synaptic growth, BDNF, and EPO levels. Specifically, this review revealed most consistent benefits of normobaric hypoxia exposures with moderate intensities between 10 and 16% O_2_, administered either intermittently or continuously, but for relatively short durations (30 min to 4 h sessions), repeatedly over 2–12 weeks. Further, most cognitive and neurological benefits occurred when the hypoxia treatments were combined with hyperoxic breathing or concurrent motor–cognitive strategies such as physical exercise or rehabilitation, possibly due to synergistic effects on neuroplastic processes. However, no definite conclusions regarding efficacy can yet be drawn given the high risk of bias in all of the human hypoxia studies due to small sample sizes, and lack of randomization and assessor blinding. Larger, methodologically stronger randomized controlled studies are thus highly warranted. If such studies replicate cognitive and neurological improvements following moderate hypoxia, this can have the potential to advance treatments targeting neuroplasticity dysfunctions and cognitive decline across CNS disorders.

## Figures and Tables

**Table 1:** Study characteristics for the included human reports (k = 10).

Author and Year	Study Design	Population	Treatment Groups	Concurrent Training	Sample Size	Age, Mean (SD)	Gender, %Female	Hypoxia Type	Exposure Type	Frequency	Intensity	Duration	Outcome Measures	Findings
Bayer et al., 2017a; 2017b; 2019 [8,9,18] *	Double-blinded RCT	Geriatric patients	Hypoxic-hyperoxia	Multimodal training intervention	*n* = 18	80.9 (7.87)	72%	Normobaric (breathing through face mask)	Intermittent	Repeated	10–14% and 30–40% O_2_	3–9 cycles of 4–7 min hypoxia and 1–2 min hyperoxia for 30–45 min, 2–3 times per week for 5–6 weeks	Dementia Detection Test and Clock Drawing Test (global cognition), 6 min walking test, Tinetti mobility test, timed-up-and-go test, and Barthel index for mobility and fall risk (motor function)	Improved performance on dementia detection test, clock drawing test, and 6 min walking test in hypoxia group compared to control group. No difference in other motor measures.
			Normoxia	Multimodal training intervention	*n* = 16	83.4 (5.5)	87.50%							
Behrendt et al., 2022 [19]	Single-blinded RCT	Geriatric patients	Hypoxia-hyperoxia	Aerobic cycling	*n* = 14	84.2 (5.1)	93%	Normobaric (breathing through face mask)	Intermittent	Repeated	10–14% and 30–40% O_2_	8–30 cycles of 1–5 min hypoxia and 1–3 min hyperoxia for 30 min, 3 times per week for 6 weeks	Dementia detection test and clock drawing test (global cognition)	Improvement on clock drawing test with medium effect size in hypoxia group compared to control group. No significant results on the dementia-detection test.
			Normoxia	Aerobic cycling	*n* = 11	85.6 (6.0)	100%							
Chen et al., 2019 [20]	Case-control study	College students	Living at high altitude		*n* = 49	19.2 (0.86)	35%	Hypobaric	Continuous	Chronic	13% O_2_	2 years of living at high altitude	Verbal memory, auditory reaction time test, structural MRI	Decreased memory performance and reaction time (within-group only; no between-group comparisons). Decreased caudate grey matter volume and higher fractional anisotropy values across various regions in high altitude group compared to control group.
			Living at low altitude		*n* = 49	19.2 (0.86)	35%							
Corral et al., 2014 [21]	Pilot study	Patients with traumatic brain injury	Hypoxia	Muscle electro-stimulation	*n* = 5	35.0 (7.0) **	0%	Hypobaric	Continuous	Repeated	12% O_2_	2 h hypoxia, 3 days per week for 12 weeks	RAVLT (memory), TMT A + B (processing speed and executive functioning), Stroop test (executive functioning), WAIS-III (global cognition), orientation, verbal fluency (executive functioning), and Tower of London (executive functioning)	No significant difference between hypoxia group and control group.
			Control group		*n* = 4	35.0 (7.0) **	0%							
Schega et al., 2013 [22]	Randomized controlled pilot study	Older adults	Hypoxia	Full-body strength-endurance training program	*n* = 17	63.7 (3.4)	76.50%	Normobaric (breathing through face mask)	Intermittent	Repeated	90% SpO_2_ week 1–2; 85% SpO_2_ week 3; 80% SpO_2_ week 4–6	4 cycles of 10 min hypoxia and 5 min normoxia for 1 h, 3 times per week for 6 weeks	MMSE (global cognition), d2 test (attention), Number Combination Test (processing speed)	Improved performance on the d2 test in hypoxia group compared to control group. No significant difference on other cognitive measures.
			Normoxia	Full-body strength-endurance training program	*n* = 17	63.6 (3.2)	76.50%							
Schega et al., 2016 [23]	Single-blinded RCT	Older adults	Hypoxia	Aerobic training	*n* = 17	66.4 (3.3)	50%	Normobaric (breathing through face mask)	Intermittent	Repeated	90–85% SpO_2_ week 1; 80% SpO_2_ week 2–4	90 min hypoxia, 3 times per week for 4 weeks	Stroop test (executive functioning)	Improved executive functioning within hypoxia group. No signficant group-by-time interactions.
			Normoxia	Aerobic training	*n* = 16	67.9 (4.4)	44.40%							
Serebrovska et al., 2019 [24]	Randomized controlled pilot study	MCI patients	Hypoxia-hyperoxia		*n* = 8	68.2 (7.2)	75%	Normobaric (breathing through face mask)	Intermittent	Repeated	12% and 33% O_2_	4 cycles of 5 min hypoxia and 3 min hyperoxia for 30 min, 5 times per week for 3 weeks	Montreal Cognitive Assessment (global cognition), EEG oddball paradigm	Improvement in global cognition and P300 and N200 latency in hypoxia group compared to normoxia group.
			Normoxia		*n* = 6	72.6 (6.9)	100%							
Serebrovska et al., 2022 [25]	Single-blinded RCT	MCI patients	Hypoxia-hyperoxia		*n* = 8	65.4 (6.2)	87.50%	Normobaric (breathing through face mask)	Intermittent	Repeated	12% and 33% O_2_	4 cycles of 5 min hypoxia and 3 min hyperoxia for 32 min, 5 times per week for 3 weeks	Montreal cognitive assessment (global cognition), EEG oddball paradigm, inflammation	Improved cognition in MCI patients after hypoxia vs. normoxia. Improved P300 and N200 in MCI patients and healthy participants after hypoxia vs. normoxia. Some evidence for increased inflammatory markers after hypoxia.
			Normoxia		*n* = 6	70.8 (9.3)	100%							
		Healthy participants	Hypoxia-hyperoxia		*n* = 6	67.5 (7.0)	83%	Normobaric (breathing through face mask)	Intermittent	Repeated	12% and 33% O_2_	4 cycles of 5 min hypoxia and 3 min hyperoxia for 32 min, 5 times per week for 3 weeks		
			Normoxia		*n* = 7	65.0 (8.1)	71%							

* Three reports from one study. ** Mean age and SD for the whole sample. Abbreviations: EEG = electroencephalography. MCI = mild cognitive impairment. MRI = magnetic resonance imaging. MMSE = mini mental state examination. O_2_ = Oxygen. RAVLT = Rey’s auditory verbal learning test. RCT = randomized controlled trial. SD = standard deviation. SpO_2_ = peripheral capillary oxygen saturation. TMT A + B = trail making test A+B. WAIS-III = Wechsler’s adult intelligence scale III.

**Table 2:** Study characteristics for the included animal reports (k = 48).

Author and Year	Animals	Intervention/Insult	Treatment Groups	Concurrent Treatment	Sample Sizes	Hypoxia Type	Exposure Type	Frequency	Intensity	Duration	Outcome Measures	Main Findings
Arnold et al., 2021 [26]	Sprague Dawley rats	Spinal cord injury	Hypoxia	Task-specific training	*n* = 16	No information	Intermittent	Repeated	11% O_2_	10 cycles of 5 min hypoxia and 5 min normoxia for 100 min, 4–7 days weekly for 12 weeks	Single pellet reaching task, Ladder walking task, and Adhesive removal task (motor function)	Some evidence of improved post-stroke motor function after hypoxia vs. normoxia.
			Normoxia	Task-specific training	*n* = 15							
Chen et al., 2021 [27]	C57BL/6 mice		Hypoxia		*n* = 12	Normobaric	Continuous	Chronic	10–11% O_2_	12 h daily for 4 weeks	Open field test (locomotor function), beam walking test (motor function), myliogenesis	Impaired motor coordination after hypoxia vs. normoxia. No significant difference in locomotor function. Inhibited myliogenesis, but no signs of myelin degeneration, after hypoxia vs. normoxia.
			Normoxia		*n* = 12							
Chung et al., 2015 [28]	Pediatric EGFP transgenic mice		Hypoxia		*n* = 5–7	Normobaric	Continuous	Chronic	10 ± 0.1% O_2_	24 h daily for 14 days	Myelination, hippocampal neurogenesis	Decreased hippocampal neurogenesis, oligodendrocyte progenitors, and mature myelin after hypoxia vs. normoxia.
			Normoxia		*n* = 5–7							
Erken et al., 2013 [29]	Wistar albino rats		Hypoxia		*n* = 7	Hypobaric	Continuous	Chronic	14% O_2_	24 h daily for 28 days	EEG recordings	Decreased delta activity coupled with increased alpha and beta activity in the three hypoxia-combination groups vs. normoxia. Decreased theta activity after hypoxia vs. normoxia.
			Hypoxia	Exercise	*n* = 7							
			Hypoxia	Docosahexaenoic acid	*n* = 7							
			Hypoxia	Exercise + docosahexaenoic acid	*n* = 7							
			Normoxia		*n* = 7							
Goldbart et al., 2003 [30]	Sprague Dawley rats		Hypoxia		*n* = 24	Normobaric	Intermittent	Chronic	10% O_2_	480 cycles of hypoxia and normoxia every 90 s for 12 h, daily for 30 days	Morris water maze task (spatial memory), hippocampal neuroapoptosis	No significant difference in spatial memory performance. No significant difference in neuroapoptosis.
			Normoxia		*n* = 24							
Gozal et al., 2010 [31]	Sprague Dawley rats		Hypoxia	Physical activity	*n* = 18	Normobaric	Intermittent	Chronic	10% O_2_	480 cycles of hypoxia and normoxia every 90 s for 12 h, daily for 14 days	Morris water maze task (spatial memory), oxidative stress	Impaired spatial memory after hypoxia+normal activity, but not hypoxia+physical activity, compared to other groups. Increased oxidative stress after hypoxia+normal activity, but not hypoxia+physical activity, compared to normoxia.
			Hypoxia	Normal activity	*n* = 18							
			Normoxia	Physical activity	*n* = 18							
			Normoxia	Normal activity	*n* = 18							
Gozal et al., 2001 [32]	Sprague Dawley rats		Hypoxia		*n* = 18	Normobaric	Intermittent	Chronic	10% O_2_	480 cycles of hypoxia and normorxia every 90 s or 24 cycles every 30 min for 12 h, daily for 14 days	Morris water maze task (spatial memory)	Some evidence of impaired spatial memory after hypoxia vs. normoxia.
			Normoxia		*n* = 19							
Gozal et al., 2003 [33]	Sprague Dawley rats		Hypoxia		*n* = 22	Normobaric	Intermittent	Chronic	10% O_2_	480 cycles of hypoxia and normoxia every 90 s for 12 h, daily for 30 days	Morris water maze task (spatial memory), hippocampal neurogenesis	No significant difference in spatial memory performance. Enhanced neurogenesis after 14–30 days hypoxia vs. normoxia.
			Normoxia		*n* = 24							
Guerra-Narbona et al. 2013 [34]	C57BL/6 mice		Hypoxia		*n* = 16	Hypobaric	Continuous	Chronic	11% O_2_	24 h daily for 14 days	Skinner box and classical eyeblink conditioning paradigm (procedural memory), Object recognition, and eight-arm radial maze task (spatial memory)	Improved learning and memory after hypoxia vs. normoxia.
			Normoxia		*n* = 16							
Hui-guo et al., 2010 [35]	Sprague Dawley rats		Hypoxia		*n* = 10	No information	Intermittent	Chronic	10% O_2_	400 cycles of 5 s hypoxia and normoxia every 90 s, 10 h daily for 4 weeks	Morris water maze task (spatial memory), oxidative stress, neuroapoptosis	Impaired spatial memory after hypoxia vs. normoxia. Increased oxidative stress and neuroapoptosis after hypoxia vs. normoxia.
			Normoxia		*n* = 10							
Ji et al., 2021 [36]	Sprague Dawley rats		High altitude		*n* = 20	Hypobaric	Continuous	Chronic	12% O_2_	24 h daily for 4–8 weeks	Morris water maze task (spatial memory), hippocampal apoptosis	Impaired spatial memory after high altitude for 8 weeks vs. sea level. Increased apoptosis after high altitude for 8 weeks vs. sea level.
			Sea level		*n* = 20							
Ju et al., 2012 [37]	Sprague Dawley rats	Ethanol withdrawal stress after 4 weeks of ethanol diet (6.5%)	Hypoxia	Ethanol withdrawal	*n* = 7–10	No information	Intermittent	Repeated	9.5–10% O_2_	5–8 cycles of 5–10 min hypoxia and 4 min normoxia, daily for 20 days	Rotarod test (motor function)	Preserved motor function in hypoxia-treated rats with ethanol withdrawal compared to normoxic-treated rats with ethanol withdrawal. No significant motor difference between hypoxia vs. normoxia only.
			Hypoxia	Sham	*n* = 7–10							
			Normoxia	Ethanol withdrawal	*n* = 7–10							
			Normoxia	Sham	*n* = 7–10							
Kheirandish et al., 2005 [38]	Pediatric Sprague Dawley rats		Hypoxia		*n* = 24	Normobaric	Intermittent	Chronic	10% O_2_	240 cycles of hypoxia and normoxia for 90 s for 12 h, daily for 20 days	Morris water maze task (spatial memory), modified working memory water maze task (spatial working memory), dendritic morphology in frontal cortex	Impaired working memory in male, but not female, rats after hypoxia vs. normoxia. Decreased dendritic branching in male, but not female, rats after hypoxia vs. normoxia.
			Normoxia		*n* = 24							
Lei et al., 2022 [39]	Sprague Dawley rats		Hypoxia		*n* = 8–12	Normobaric	Continuous	Repeated	10% O_2_	6 h daily for 1 month	Open field test (locomotor function), novel object recognition and Morris water maze task (spatial memory), tau hyperphosphorylation	Impaired spatial memory after hypoxia vs. normoxia. No significant difference in locomotor function. Increased tau hyperphosphorylation after hypoxia vs. normoxia.
			Normoxia		*n* = 8–12							
Li et al., 2022 [40]	C57BL mice		Continuous hypoxia		*n* = 10	No information	Continuous	Chronic	13% O_2_	24 h daily for 14 days	Novel object recognition test and Morris water maze task (spatial memory), hippocampal neuroapoptosis	Impaired spatial memory after 14 days of chronic hypoxia, but also after reoxygeneration (hypoxia+normoxia group). Some evidence for impaired spatial memory after intermittent hypoxia preconditioning vs. normoxia. Neurodegeneration after chronic hypoxia vs. normoxia.
			Intermittent hypoxia + continuous hypoxia		*n* = 10		Intermittent	Repeated followed by chronic	13% O_2_	10 cycles of 5 min hypoxia and 5 min normoxia for 100 min, daily for 14 days, followed by 24 h daily for 14 days		
			Continuous hypoxia + normoxia		*n* = 10		Continuous	Chronic	13% and 21% O_2_	24 h daily for 14 days followed by normoxia 24 h daily for 28 days		
			Normoxia		*n* = 10							
Liu et al., 2016 [41]	APPswe/PS1dE9 mice		Hypoxia		*n* = 30	Hypobaric	Continuous	Repeated	11.1% O_2_	6 h daily for 30 days	Morris water maze test (spatial memory), β-amyloid, synaptic morhphology in hippocampus	Impaired spatial memory after hypoxia vs. normoxia group. Loss of synapses and elevated β-amyloid after hypoxia vs. normoxia.
			Normoxia		*n* = 30							
Lu et al., 2009 [42]	Prediatric ICR mice		Hypoxia		*n* = 14	Hypobaric	Continuous	Repeated	16% O_2_	4 h daily for 4 weeks	Morris water maze test and Eight-arm radial maze (spatial memory), LTP in hippocampus	Improved spatial memory after hypoxia vs. normoxia. Enhanced hippocampal LTP after hypoxia vs. normoxia.
			Normoxia		*n* = 14							
Manukhina et al., 2010 [43]	Wistar rats	AD model induced by β-amyloid administration	Hypoxia		No information	Hypobaric	Continuous	Repeated	12.5% O_2_	4 h daily for 14 days	Conditioned passive avoidance reflex (procedural memory), oxidative stress, cortical neuroapoptosis	Improved memory and no neuroapoptosis in hypoxia+β-amyloid group vs. normoxia+β-amyloid group. No difference between non-injected hypoxia and normoxia group. No neuroapoptosis in hypoxia+β-amyloid group vs. normoxia+β-amyloid group. No significannt difference in oxidative stress.
			Hypoxia	β-amyloid	No information							
			Normoxia		No information							
			Normoxia	β-amyloid	No information							
Manukhina et al., 2018 [44]	Wistar rats	PTSD model of predator stress	Hypoxia		*n* = 20	Hypobaric	Continuous	Repeated	12.5% O_2_ (20% to 12.5% O_2_ for first 5 days)	4 h daily for 14 days (0.5–3 h for the first 5 days)	Elevated plus-maze test (emotional reactivity)	Less anxiety-related behavior after hypoxia vs. normoxia. Hypoxia-treated PTSD rats had less anxiety-related behavior than normoxic PTSD rats.
			Hypoxia	PTSD	*n* = 20							
			Normoxia		*n* = 20							
			Normoxia	PTSD	*n* = 20							
Meng et al., 2020 [45]	APP/PS1 mice		Hypoxia		*n* = 12	Hypobaric	Continuous	Repeated	11% O_2_	4 h daily for 15 days	Morris water maze test (spatial memory), novel object recognition test, spontaneuous alternation Y-maze test, open field test and elevated plus-maze test (locomotor function, exploratory and anxiety-related behavior), hippocampal neurogenesis, BDNF	Improved memory and locomotor function after hypoxia vs. normoxia. Enhanced neurogenesis and BDNF levels in hippocampus after hypoxia vs. normoxia.
			Normoxia		*n* = 12							
Ortega et al., 2016 [46]	Pediatric CD1 mice		Hypoxia		*n* = 10	No information	Continuous	Chronic	10 ± 0.1% O_2_	24 h daily for 25 days	Rotarod test and force grip test (motor function), myelination, neuroinflammation	Some evidence for motor impairment at 4 weeks after hypoxia vs. normoxia. Reduced mature myelin after hypoxia vs. normoxia, which persisted 4 weeks after hypoxia treatment. Increased inflammation after hypoxia vs. normoxia.
			Normoxia		*n* = 10							
Perry et al., 2008 [47]	Wistar Hannover rats		Hypoxia		*n* = 12	Normobaric	Intermittent	Repeated	10% O_2_	6 h daily for 21 days	Locomotor-recording chamber and inhibitory avoidance task (motor function)	Increased locomotor activity in hypoxia+sleep restriction group vs. normoxia group. No significant difference in motor function.
			Hypoxia	Sleep restriction	*n* = 12							
			Normoxia	Sleep restriction	*n* = 12							
			Normoxia		*n* = 12							
Pietrogrande et al., 2019 [48]	Non-specified mice	Stroke induced by vascular occlusion	Hypoxia		*n* = 9	Normobaric	Continuous	Repeated	11% O_2_	8 h daily for 14 days	Cylinder test and grid walk test (motor function), BDNF	Improved post-stroke motor function in hypoxia-treated group vs. normoxia group, which persisted 2 weeks after treatment. Increased BDNF levels 2 weeks after hypoxia vs. normoxia.
			Normoxia		*n* = 7							
Row et al., 2007 [49]	Sprague Dawley rats		Hypoxia		*n* = 8	Normobaric	Intermittent	Chronic	10% O_2_	312 cycles of hypoxia and normoxia every 90 s for 12 h, daily for 14 days	Delayed matching to sample task (working memory)	Impaired working memory after hypoxia vs. normoxia.
			Normoxia		*n* = 8							
Row et al., 2002 [50]	Pediatric Sprague Dawley rats		Hypoxia		*n* = 35	Normobaric	Intermittent	Chronic	10% O_2_	960 cycles of hypoxia and normoxia every 90 s for 24 h, daily for 14 days	Morris water maze test (spatial memory) and open field test (locomotor function)	Impaired spatial memory after hypoxia vs. normoxia. Increased locomotor activity in male, but not female, rats exposed to hypoxia vs. normoxia.
			Normoxia		*n* = 35							
Ryou et al., 2021 [51]	C57BL/B6 mice	AD (transgenic model)	Hypoxia		*n* = 6	Normobaric	Intermittent	Repeated	10% O_2_	10 cycles of 6 min hypoxia and 4 min normoxia for 1 h, daily for 21 days	Morris water maze test (spatial memory). Cerebrocortical BDNF and EPO	No significant difference in spatial memory. Increased BDNF and EPO after hypoxia vs. normoxia.
			Normoxia		*n* = 6							
Sakr et al., 2015 [52]	Sprague Dawley rats		Hypoxia		*n* = 32	No information	Continuous	Chronic	12% O_2_	24 h daily for 8 weeks	Cortical BDNF, oxidative stress	Increased oxidative stress and BDNF levels after hypoxia vs. normoxia.
			Normoxia		*n* = 32							
Satriotomo et al., 2016 [53]	Sprague Dawley rats		Hypoxia		*n* = 10	Normobaric	Intermittent	Repeated	10.5% O_2_	10 cycles of 5 min hypoxia and 5 min normoxia for 100 min, 3 times weekly for 10 weeks	BDNF and VEGF	Increased levels of BDNF, VEGF, and their receptors after hypoxia vs. normoxia.
			Normoxia		*n* = 10							
Sharma et al., 2019 [54]	C57BL/6 mice		Hypoxia		*n* = 18	Hypobaric	Continuous	Chronic	11% O_2_	24 h daily for 12 weeks	Cued and contextual fear memory test (memory), myelination, synaptic density in hippocampus	Some evidence for impaired learning after hypoxia vs. normoxia. No signficiant difference in synaptic density or protein levels in hippocampus. Some evidence for lower levels of synaptic and astroglial proteins in the olfactory cortex, cerebellum, and brainstem, but no difference in myelin protein expression between the groups.
			Normoxia		*n* = 18							
Sun et al., 2019 [55]	Sprague Dawley rats	Pilocarpine-induced epilepsy	Hypoxia	Epilepsy	*n* = 9	Hypobaric	Continuous	Repeated	14.5% O_2_	6 h daily for 28 days	Morris water maze (spatial memory), hippocampal neuron survival and neurogenesis, neurotrophin in hippocampus and cortex	Improved memory in hypoxia+epilepsy group vs. normoxia+epilepsy group. Diminished neuron loss and increased neurogenesis in hippocampus in hypoxia+epilepsy group vs. normoxia+epilepsy group. Upregulated NT-3 and BDNF in hippocampus and temporal lobe in hypoxia+epilepsy group vs. normoxia+epilepsy group.
			Normoxia	Epilepsy	*n* = 7							
			Normoxia		*n* = 8							
Sun et al., 2021 [56]	Sprague Dawley rats	Pilocarpine-induced epilepsy	Hypoxia	Epilepsy	*n* = 12	Hypobaric	Continuous	Repeated	14% O_2_	6 h daily for 28 days	Morris water maze (spatial memory), novel object recognition test (non-spatial short-term memory), neuron survival and neurogenesis in hippocampus, dendritic morphology and synaptic ultrastructure in hippocampus.	Improved memory and object recognition in hypoxia+epilepsy group vs. normoxia+epilepsy group. Less severe neuronal loss, increased neurogenesis and synaptic plasticity, and improved dendritic structural plasticity in the hippocampus after hypoxia vs. normoxia, although these effects were absent in hypoxia+DKK-1 group.
			Hypoxia	Epilepsy + DKK-1	*n* = 11							
			Normoxia	Epilepsy	*n* = 12							
			Normoxia		*n* = 11							
Tang et al., 2020 [57]	SPF C57BL/6 mice		Hypoxia		*n* = 12	Normobaric	Intermittent	Repeated	10% O_2_	320 cycles of 30 s hypoxia and 60 s normoxia for 8 h, daily for 4 weeks	Morris water maze (spatial memory), Open field test (locomotor function and anxiety), apoptosis, oxidative stress, inflammation in the basal forebrain	Impaired spatial memory, diminished and more anxious locomotor behavior after hypoxia vs. normoxia. Increased apoptosis, oxidative stress, and inflammation in basal forebrain after hypoxia vs. normoxia.
			Normoxia		*n* = 12							
Wakhloo et al., 2020 [58]	Wildtype mice		Hypoxia		*n* = 16	No information	Continuous	Chronic	12% O_2_	24 h daily for 3 weeks	Complex running wheel (motor function)	Improved motor function after hypoxia vs. normoxia.
			Normoxia		*n* = 15							
Wang et al., 2021 [59]	C57BL/6J mice		Hypoxia		*n* = 12	No information	Intermittent	Repeated	10% O_2_	120 cycles of 2 min hypoxia and 2 min normoxia for 8 h, daily for 4 weeks	Morris water maze (spatial memory), inflammation	Impaired spatial learning after hypoxia vs. normoxia. Increased inflammation after hypoxia vs. normoxia.
			Normoxia		*n* = 12							
Warrington et al., 2012 [60]	C57BL/6 male mice	Whole brain radiation therapy	Hypoxia	Sham	*n* = 9	Normobaric	Continuous	Chronic	11% O_2_	24 h daily for 28 days	Barnes maze (spatial memory), working memory version of Barnes maze (working memory), hippocampal vascular density	Improved memory in hypoxia+radiation group vs. normoxia+radiation group. Improved working memory in hypoxia+radiation group vs. normoxia+sham group 2 months after hypoxia. No differences in cognitive performance between hypoxia+sham group and normoxia+sham group. Increased hippocampal vascular density in both hypoxia groups after 2 months.
			Hypoxia	Whole brain radiation	*n* = 9							
			Normoxia	Sham	*n* = 9							
			Normoxia	Whole brain radiation	*n* = 9							
Xu et al., 2015 [61]	C57BL/6J mice		Hypoxia		*n* = 4–10	Normobaric	Intermittent	Repeated	10% O_2_	320 cycles of hypoxia and normoxia every 90 s for 8 h, daily for 14 days	Radial arm maze test and object recognition task (spatial working memory and long-term memory), open field test (locomotor function and anxiety), apoptosis, hippocampal synaptic plasticity	Some evidence for memory impairment after 14 days of hypoxia vs. normoxia. No statistical difference in locomotor activity and anxiety. Increased apoptosis and decreased synaptic plasticity in the hippocampus after hypoxia vs. normoxia. No statistical difference in number of excitatory synapses in the hippocampus.
			Normoxia		*n* = 4–10							
Yang et al., 2012 [62]	Geriatric Sprague Dawley rats		Hypoxia		*n* = 10	No information	Intermittent	Repeated	10% O_2_	320 cycles of 5 s hypoxia and 85 s normoxia for 8 h, daily for 4 weeks	Morris water maze (spatial memory), hippocampal apoptosis	Impaired memory performance after hypoxia vs. normoxia. Increased apoptosis in the hippocampus in hypoxia groups vs. normoxia.
			Hypoxia	N-acetylcystein	*n* = 10							
			Normoxia		*n* = 10							
			Normoxia	N-acetylcystein	*n* = 10							
Yu et al., 2016 [63]	Kunming mice		Hypoxia		*n* = 10	Hybobaric	Continuous	Repeated	10.5% O_2_	8 h daily for 28 days	Radial arm maze (spatial working memory and long-term memory), step-through passive avoidance test (learning and memory), tau phosphorylation	Impaired learning, memory and working memory after hypoxia vs. normoxia. Elevated tau phosphorylation after hypoxia vs. normoxia.
			Normoxia		*n* = 10							
Yuan et al., 2019 [64]	Sprague Dawley rats		Hypoxia		*n* = 8–16	Hypobaric	Continuous	Chronic	10% O_2_	4 weeks	Morris water maze (spatial memory), resting-state fMRI	Impaired memory and decreased resting-state activity across the brain after hypoxia vs. normoxia.
			Normoxia		*n* = 8–16							
Yue et al., 2021 [65]	C57BL/6J wild-type mice		Hypoxia		*n* = 10–12	Hypobaric	Continuous	Repeated	14.3% O_2_	4 h daily for 14 days	Morris water maze (spatial memory), amyloid plaque, neuroinflammation	Improved memory after hypoxia+AD vs. normoxia+AD after both 14 and 28 days of hypoxia treatment, which dissapeared at 42-day follow-up (for 14 days hypoxia). No statistical difference in memory in wild-type mice after hypoxia vs. normoxia. Decreased amyloid plaque in hippocampus, but not in cortex, and reduced inflammation after hypoxia+AD (14 days hypoxia) vs. normoxia+AD.
	APP/PS1 mice	AD	Hypoxia		*n* = 10–12					4 h daily for 14 days		
	C57BL/6J wild-type mice		Hypoxia		*n* = 10–12					4 h daily for 28 days		
	APP/PS1 mice	AD	Hypoxia		*n* = 10–12					4 h daily for 28 days		
	C57BL/6J wild-type mice		Normoxia		*n* = 10–12							
	APP/PS1 mice	AD	Normoxia		*n* = 10–12							
Zhang et al., 2014 [66]	Sprague Dawley rats		Hypoxia		*n* = 12	No information	Continuous	Repeated	10% O_2_	6 h, 6 days per week for 2 weeks	Morris water maze (spatial memory), tau phosphorylation, oxidative stress.	Impaired memory after all durations of hypoxia vs. normoxia. Increased hippocampal tau phosphorylation and oxidative stress after all durations of hypoxia vs. normoxia.
			Hypoxia		*n* = 12					6 h, 6 days per week for 4 weeks		
			Hypoxia		*n* = 12					6 h, 6 days per week for 6 weeks		
			Normoxia		*n* = 12							
Zhang et al., 2006 [67]	ICR mice		Hypoxia		*n* = 8	Hypobaric	Continuous	Repeated	10.8% O_2_	4 h daily for 15 days	Morris water maze (spatial memory), shuttle-box test (non-declarative memory), open field test (locomotor function and anxiety).	Impaired non-declarative memory after 25 days of 10.8% hypoxia (but not 16%) vs. normoxia. No statistical difference in locomotor activity and anxiety and spatial memory after 25 days of 10.8% or 16% hypoxia vs. normoxia. No results reported on 15-day hypoxia group.
			Hypoxia		*n* = 7–8				10.8% O_2_	4 h daily for 25 days		
			Hypoxia		*n* = 7–8				16% O_2_	4 h daily for 15 days		
			Hypoxia		*n* = 8				16% O_2_	4 h daily for 25 days		
			Normoxia		*n* = 7–8							
Zhang et al., 2005 [68]	Neonatal ICR mice		Hypoxia		*n* = 7	Hypobaric	Continuous	Repeated	10.8% O_2_	4 h daily for 2 weeks	Morris water maze and 8-arm maze (spatial memory), open field test (locomotor function and anxiety), LTP and synaptic density in hippocampus.	Improved spatial memory in after 3 and 4 weeks of hypoxia (but not 2 weeks) vs. normoxia, which persisted at 3 months follow-up for the 4-week 16% O_2_ group. Improved locomotor function after 4-week hypoxia vs. normoxia. Increased hippocampal synapses and LTP after 4-week hypoxia vs. normoxia.
			Hypoxia		*n* = 7				10.8% O_2_	4 h daily for 3 weeks		
			Hypoxia		*n* = 7				10.8% O_2_	4 h daily for 4 weeks		
			Hypoxia		*n* = 7				16% O_2_	4 h daily for 2 weeks		
			Hypoxia		*n* = 7				16% O_2_	4 h daily for 3 weeks		
			Hypoxia		*n* = 7				16% O_2_	4 h daily for 4 weeks		
			Normoxia		*n* = 7							
Zhang et al., 2022 [69]	C57BL/6J wild-type mice		Hypoxia		*n* = 11	Hypobaric	Continuous	Chronic	10% O_2_	23 h daily for 4 weeks	Morris water maze (spatial memory), fear conditioning test, hippocampal neuroapoptosis	Impaired memory and contextual and cued fear memory after hypoxia vs. normoxia. Increased neuronal damage after hypoxia vs. normoxia.
			Normoxia		*n* = 11							
Zhao et al., 2022 [70]	C57BL/6 mice		Hypoxia		*n* = 12	Hypobaric	Continuous	Chronic	12.5–13.5% O_2_	14 days	Novel object test (working memory), EEG P300 in oddball behavioral paradigm training test, apoptosis in the prefrontal cortex	Impaired working memory capacity and decreased P300 amplitude (indicating working memory deficits) in both hypoxia groups vs. normoxia. Some evidence of increased apoptosis in prefrontal cortex in hypoxia groups vs. normoxia.
			Hypoxia	Antibiotics	*n* = 12							
			Normoxia		*n* = 12							
Zhu et al., 2022 [71]	Sprague Dawley rats		Living at high altitude		*n* = 6–12	Hypobaric	Continuous	Chronic	12.5% O_2_	8 months of living at high altitude	Morris water maze (spatial memory), open field test and elevated plus maze (locomotor function and anxiety), structural MRI of the hippocampus, oxidative stress in the hippocampus and cortex, inflammation in the hippocampus and cortex, hippocampal neurodegeneration	Impaired memory and higher anxiety levels after hypoxia vs. normoxia. Reduced hippocampal volume after hypoxia, which correlated with poorer memory. Increased oxidative stress and inflammation in the hippocampus and cortex after hypoxia vs. normoxia. Increased hippocampal neurodegeneration after hypoxia vs. normoxia.
			Living at low altitude		*n* = 6–12							
Zhu et al., 2021 [72]	C57BL/6 mice		Hypoxia		*n* = 8	Normobaric	Intermittent	Repeated	10% O_2_	320 cycles of 30 s hypoxia and 60 s normoxia for 8 h, daily for 4 weeks	Morris water maze (spatial memory), hippocampal apoptosis, oxidative stress	Impaired memory after hypoxia vs. normoxia. Increased hippocampal apoptosis and oxidative stress after hypoxia vs. normoxia.
			Normoxia		*n* = 8							
Zhu et al., 2010 [73]	Sprague Dawley rats and albino Wistar rats		Hypoxia		*n* = 6–10	Hypobaric	Continuous	Repeated	11.2% O_2_	4 h daily for 14 days	Forced swimming test, chronic mild stress test, and novelty suppressed feeding test (depressive behavior), open field test (locomotor function and anxiety), hippocampal neurogenesis and apoptosis, EPO, hippocampal BDNF	Decreased depressive behavior in 11.2% O_2_ group (and not in 14.4% O_2_ group). No group differences in locomotor function (open field test). Enhanced neurogenesis after 11.2% O_2_ (but not 14.4% O_2_) vs. normoxia. Increased BDNF, EPO, and no neuroapoptosis after 11.2% O_2_ vs. normoxia.
			Hypoxia		*n* = 6–10	Hypobaric	Continuous	Repeated	14.4% O_2_	4 h daily for 14 days		
			Normoxia		*n* = 6–10							

Abbreviations: AD = Alzheimer’s disease. BDNF = brain-derived neurotrophic factor. DKK-1 = Wnt/β-catenin antagonist Dickkopf-1. EEG = electroencephalography. EPO = Erythropoietin. fMRI = functional magnetic resonance imaging. LTP = long-term potentiation. NT-3 = Neurotrophin-3. PTSD = posttraumatic stress disorder. O_2_ = Oxygen. VEGF = vascular endothelial growth factor.

**Table 3:** Cochrane risk of bias (RoB) 2.0 in human intervention studies.

Study	Domain 1	Domain 2	Domain 3	Domain 4	Domain 5	Overall Risk of Bias
Bayer et al., 2017a; 2017b; 2019 * [8,9,18]	Some concerns	Some concerns	Some concerns	Low risk	Some concerns	High risk
Behrendt et al., 2022 [19]	Low risk	Some concerns	Some concerns	High risk	Low risk	High risk
Corral et al., 2014 [21]	High risk	High risk	Some concerns	High risk	Low risk	High risk
Schega et al., 2013 [22]	Low risk	Some concerns	Low risk	High risk	Some concerns	High risk
Schega et al., 2016 [23]	Some concerns	Some concerns	Some concerns	High risk	Some concerns	High risk
Serebrovska et al., 2019 [24]	Some concerns	Some concerns	Some concerns	High risk	Some concerns	High risk
Serebrovska et al., 2022 [25]	Some concerns	Some concerns	Low risk	High risk	Some concerns	High risk

Notes: * = Three reports of the same study. Domain 1: risk of bias arising from the randomization process. Domain 2: risk of bias due to deviations from the intended interventions (effect of assignment to intervention). Domain 3: risk of bias due to missing outcome data. Domain 4: risk of bias in measurement of the outcome. Domain 5: risk of bias in selection of the reported results.

**Table 4:** Risk of bias in nonrandomized studies—of interventions (ROBINS-I) in human observational studies.

Study	Domain 1	Domain 2	Domain 3	Domain 4	Domain 5	Domain 6	Domain 7	Overall Risk of Bias
Chen et al., 2019 [20]	Serious risk	Serious risk	Low risk	Low risk	Moderate risk	Serious risk	No information	Serious risk

Notes: Domain 1: bias due to confounding. Domain 2: bias in selection of participants into the study. Domain 3: bias in classification of interventions. Domain 4: bias due to deviations from intended interventions. Domain 5: bias due to missing data. Domain 6: bias in measurement of outcomes. Domain 7: bias in selection of the reported result.

## Supplementary Materials

The following supporting information can be downloaded at: https://www.mdpi.com/article/10.3390/brainsci13121648/s1. Ref. [89] is cited in the Supplementary Materials.
