# Supplementary material for: Neuroprotective Effects of Moderate Hypoxia: A Systematic Review
Source: Brain Sci. 2023 Nov 27;13(12):1648. doi: 10.3390/brainsci13121648 (PMC10741927; doi:10.3390/brainsci13121648)
Supplement: Supplementary file 1 [file brainsci-13-01648-s001.zip › Search string May 2023.pdf]

Search protocol

Search profile on PubMed – 2nd May 2023

	Focus: Hypoxia	Focus: Cognition and neurological outcome	Focus: Intervention	Exclusion criteria		
MeSH	#1	#2		#3	#4	**3536 hits**
Ti/Ab	#5	#6	#7	#8	#9	**863 hits**
NOT MEDLINE [SB]					#10	**151 hits**

Search lines PubMed

Search #1: “Hypoxia”[Mesh] OR “Altitude[Mesh]

Search #2: “Cognition”[Mesh] OR “Executive Function”[Mesh] OR “Memory”[Mesh] OR “Motor Activity”[Mesh]

Search #3: NOT (“Ischemia”[Mesh] OR “Review”[PT])

Search #4: (“Hypoxia”[Mesh] OR “Altitude”[Mesh]) AND (“Cognition”[Mesh] OR “Executive Function”[Mesh] OR “Memory”[Mesh] OR “Motor Activity”[Mesh]) NOT (“Ischemia”[Mesh] OR “Review”[PT])

Search #5: “hypoxia”[Title/Abstract] OR “altitude”[Title/Abstract]

Search #6: “cogni*”[Title/Abstract] OR “executive function*”[Title/Abstract] OR “memor*”[Title/Abstract] OR “attention*”[Title/Abstract] OR “walk*”[Title/Abstract] OR “spinal cord”[Title/Abstract]

Search #7: “treat*”[Title/Abstract] OR “interven*”[Title/Abstract] OR “daily”[Title/Abstract] OR “weekly”[Title/Abstract]

Search #8: NOT (“ischemi*”[Title/Abstract] OR “Review”[PT])

Search #9: (“hypoxia”[Title/Abstract] OR “altitude”[Title/Abstract]) AND (“cogni*”[Title/Abstract] OR “executive function*”[Title/Abstract] OR “memor*”[Title/Abstract] OR “walk*”[Title/Abstract] OR “spinal cord”[Title/Abstract]) AND (“treat*”[Title/Abstract] OR “interven*”[Title/Abstract]) NOT (“ischemi*”[Title/Abstract] OR “Review”[PT])

Search #10: (“hypoxia”[Title/Abstract] OR “altitude”[Title/Abstract]) AND (“cogni*”[Title/Abstract] OR “executive function*”[Title/Abstract] OR “memor*”[Title/Abstract] OR “walk*”[Title/Abstract] OR “spinal cord”[Title/Abstract]) AND (“treat*”[Title/Abstract] OR “interven*”[Title/Abstract]) NOT (“ischemi*”[Title/Abstract] OR “Review”[PT] OR “MEDLINE”[Filter])

Limits: English language

Search profile on PsycInfo – 2nd May 2023

	Focus: Hypoxia	Focus: Cognition and neurological outcome	Focus: Intervention	Exclusion criteria		
Ti/Ab	#1	#2	#3	#4	#5	**275 hits**

Search lines PsycInfo

Search #1: AB hypoxia OR TI hypoxia

Search #2: AB cogniti* OR AB executive function* OR AB memory OR AB walk* OR AB perception OR AB attention OR TI cogniti* OR TI executive function* OR TI memory OR TI walk* OR TI perception OR TI attention

Search #3: AB interven* OR AB treat* OR TI interven* OR TI treat*

Search #4: NOT (AB ischemi* OR TI ischemi*)

Search #5: (AB hypoxia OR TI hypoxia) AND (AB cogniti* OR AB executive function* OR AB memory OR AB walk* OR AB spinal cord OR AB perception OR AB attention OR TI cogniti* OR TI executive function* OR TI memory OR TI walk* OR TI spinal cord OR TI perception OR TI attention) AND (AB interven* OR AB treat* OR TI interven* OR TI treat*) NOT (AB ischemi* OR TI ischemi*)

Limits: English language

Search profile on EMBASE & Cochrane library – 2nd May 2023

	Focus: Hypoxia	Focus: Cognition and neurological outcome	Focus: Intervention	Exclusion criteria		
EMTree	#1	#2	#3	#4	#5	**668 hits**
Ti/Ab	#6	#7	#8	#9	#10	**1414 hits**

Search lines EMBASE

Search #1: exp hypoxia/

Search #2: cognition/ or walking/ or spinal cord/ or memory/ or executive function/

Search #3: exp therapy/ or exp intervention study/

Search #4: not (exp ischaemia/)

Search #5: (exp hypoxia/) AND (cognition/ or walking/ or spinal cord/ or memory/ or executive function/) AND (exp therapy/ or exp intervention study/) NOT (exp ischaemia/)

Search #6: hypoxia.ab. or hypoxia.ti.

Search #7: cogniti*.ab. or cogniti*.ti. or spinal cord.ab. or spinal cord.ti. or memory.ab. or memory.ti. or executive function*.ab. or executive function*.ti.

Search #8: treat*.ab. or treat*.ti. or intervention.ab. or intervention.ti.

Search #9: not (ischemi*.ab or ischemi*.ti)

Search #10: (hypoxia.ab. or hypoxia.ti.) and (cogniti*.ab. or cogniti*.ti. or spinal cord.ab. or spinal cord.ti. or memory.ab. or memory.ti. or executive function*.ab. or executive function*.ti.) and (treat*.ab. or treat*.ti. or intervention.ab. or intervention.ti.) NOT (ischemi*.ab or ischemi*.ti)

Limits: English language
